# Identification of bipolar disorder using a combination of multimodality magnetic resonance imaging and machine learning techniques

**DOI:** 10.1186/s12888-020-02886-5

**Published:** 2020-10-06

**Authors:** Hao Li, Liqian Cui, Liping Cao, Yizhi Zhang, Yueheng Liu, Wenhao Deng, Wenjin Zhou

**Affiliations:** 1 Department of Neurology, The First Affiliated Hospital, Sun Yat-sen University, Guangzhou, China; 2 Guangdong Provincial Key Laboratory of Diagnosis and Treatment of Major Neurological Diseases, National Key Clinical Department and Key Discipline of Neurology, No.58 Zhongshan Road 2, Guangzhou, 510080 China; 3 Affiliated Brain Hospital of Guangzhou Medical University, Guangzhou Huiai Hospital, Guangzhou, Guangdong China; 4 Department of Psychiatry, The Second Xiangya Hospital, Central South University, Changsha, Hunan China; 5 Chinese National Clinical Research Center on Mental Disorders (Xiangya), Changsha, Hunan China

**Keywords:** Bipolar disorder, Multimodality magnetic resonance imaging, Support vector machine

## Abstract

**Background:**

Bipolar disorder (BPD) is a common mood disorder that is often goes misdiagnosed or undiagnosed. Recently, machine learning techniques have been combined with neuroimaging methods to aid in the diagnosis of BPD. However, most studies have focused on the construction of classifiers based on single-modality MRI. Hence, in this study, we aimed to construct a support vector machine (SVM) model using a combination of structural and functional MRI, which could be used to accurately identify patients with BPD.

**Methods:**

In total, 44 patients with BPD and 36 healthy controls were enrolled in the study. Clinical evaluation and MRI scans were performed for each subject. Next, image pre-processing, VBM and ReHo analyses were performed. The ReHo values of each subject in the clusters showing significant differences were extracted. Further, LASSO approach was recruited to screen features. Based on selected features, the SVM model was established, and discriminant analysis was performed.

**Results:**

After using the two-sample t-test with multiple comparisons, a total of 8 clusters were extracted from the data (VBM = 6; ReHo = 2). Next, we used both VBM and ReHo data to construct the new SVM classifier, which could effectively identify patients with BPD at an accuracy of 87.5% (95%CI: 72.5–95.3%), sensitivity of 86.4% (95%CI: 64.0–96.4%), and specificity of 88.9% (95%CI: 63.9–98.0%) in the test data (*p* = 0.0022).

**Conclusions:**

A combination of structural and functional MRI can be of added value in the construction of SVM classifiers to aid in the accurate identification of BPD in the clinic.

## Background

Bipolar disorder (BPD) is a chronic and disabling mood disorder found in up to 2.5% of the population. It is characterized by extreme fluctuations in mood, functionality, and energy, in addition to recurrent depressive and manic/hypomanic episodes. Due to the early onset of the disease, high rates of self-inflicted injury and hospitalization, and the negative stigma of BPD, the disease causes significant social and economic burden [1, 2]. It was previously reported that the risk of suicide was 20-times higher in patients with BPD than the more general population [3–6]. In addition, the clinical symptoms of BPD overlap with those of many other mood disorders, including major depressive disorder (MDD), schizophrenia, and attention deficit and hyperactivity disorder (ADHD) [7]. Due to the similar symptom profiles, BPD often goes undiagnosed or misdiagnosed for extended periods. In some cases, it may take up to 10 years after initially seeking treatment to be correctly diagnosed with BPD [8]. This further aggravates the effective treatment of BPD and results in increased disease burden. Hence, researchers are seeking new potential biomarkers to assist the diagnosis and therapeutic monitoring of BPD. Among the new biomarkers, neuroimaging biomarkers have shown excellent potential.

Magnetic resonance imaging (MRI) is a non-invasive neuroimaging technique used to assess the internal anatomy of the brain. In recent years, MRI has been extensively utilized in neuroimaging studies as a potential biomarker. For structural MRI, voxel-based morphometry (VBM) is one of the most common techniques used to assess focal differences in brain anatomy. The MRI scans of individuals are normalized to a standard template, and voxel-by-voxel comparisons are used to investigate localized abnormalities in gray matter density or volume [9]. For functional MRI, regional homogeneity (ReHo) is a data-driven and highly established approach to evaluate local activity in the brain while at rest. In practice, ReHO represents the temporal homogeneity of the regional blood oxygen level-dependent (BOLD) signal by using Kendall’s coefficient of concordance (KCC), which is a number from 0 to 1 that indicates interrater agreement [10]. Fluctuations in ReHo are indicative of local abnormalities in brain activity [11]. Through both VBM and ReHo studies, BPD has been identified as a disorder with several morphological and functional brain abnormalities. However, there have been some inconsistencies between the studies. In terms of VBM studies, abnormalities have been detected extensively throughout the brain, including the frontal lobe, temporal lobe, parietal lobe, cingulate cortex, and the cerebellum. In addition, some of these findings have contradicted each other. For example, some studies shown increased gray matter volumes in the ventral prefrontal cortex (PFC) [12], inferior frontal gyrus [13, 14], middle temporal gyri and left temporal pole [15], cingulate gyrus [12, 16], putamen [17, 18], and cerebellum [18], while other studies found reduced gray matter volumes in the same areas [19–27]. A similar situation exists with the ReHo analysis. Some studies found increased ReHo values in the frontal lobe, cingulate cortex, and parahippocampal gyrus, while others found reduced ReHo values in the same areas [28–32]. As most of the studies mentioned above were performed at the group level, it is challenging to apply these findings to the individual identification of BPD.

Recently, machine learning approaches have introduced to address the dilemma of inconsistencies. In machine learning, the nature of the “diagnosis” is a classification problem. Among them, Support Vector Machines (SVM) have been developed from the theory of limited samples Statistical Learning Theory by Vapnik et al., which was originally designed for binary classification [33]. It is constructed based on the simplicity of structural risk minimization instead of empirical risk minimization. This enables SVM an optimal generalization ability in difficult situations [34, 35]. For these reasons, it has been widely used in the detection of mental disorders. For structural MRI, SVM has been used to accurately identify Alzheimer’s disease, autism spectrum disorders MDD, obsessive-compulsive disorder, and schizophrenia [36–40]. SVM has also been used to accurately identify different mental disorders in functional MRI studies [41–43]. In terms of BPD, Redlich and colleagues previously used an SVM algorithm based on the whole-brain gray matter to distinguish between BPD and unipolar depression with an accuracy of almost 76% [44]. In another study, SVM was used in combination with thalamic seed-based connectivity to differentiate between BPD and healthy controls (HC) with an accuracy of 61.7% [45].

However, the majority of previous studies have used single-modality MRI, either structural or functional MRI. However, a single imaging modality only provides a limited snapshot of the brain in terms of structure or function, while the combined structure-function analysis may provide a more comprehensive perspective of the brain. In recent years, multimodality MRI has been applied to SVM for the classification of schizophrenia and ASD, and the findings have verified that multimodality imaging is significantly more accurate than single modality imaging [46, 47]. Hence, in this study, we have constructed an SVM model, with VBM and ReHo measurement in gray matter volumes as features, to differentiate between patients with BPD from the HCs. We evaluated the classification capabilities of the model and identified the brain areas critical for discriminating between BPD and the HCs. To the best of our knowledge, this is the first study to distinguish between patients with BPD and HCs using an SVM classifier based on the combination of ReHo and VBM analyses.

## Methods

### Participants

Between January 2012 and December 2015, 44 patients with BPD and 36 age- and sex-matched HCs were recruited from the Affiliated Brain Hospital of Guangzhou Medical University (Guangzhou Huiai Hospital, Guangdong, China) and surrounding communities, respectively. The patients were preliminarily diagnosed as having BPD by one or more of our senior psychiatrists, based on the criteria outlined by the Diagnostic and Statistical Manual of Mental Disorders 4th Edition (DSM-IV) and the structured clinical interview for DSM-IV (SCID) for further confirmation. The HCs were also screened with SCID to ensure they had a fit mental status. All participants were from the Han population, right-handed, and had intelligence quotient scores above 75. The exclusion criteria for the BPD and HC groups were as follows: (1) comorbid Axis I or Axis II disease; (2) history of other psychiatric or neurological illness or severe physical illness; (3) active substance abuse or addiction; (4) unable to complete MRI session due to physical or mental limitations; (5) organic brain lesions detecting by MRI. The study was approved by institutional review boards of Guangzhou Huiai Hospital, and written informed consent was obtained from each participant or their legal guardians before the study.

### Collection of demographic and clinical information

General demographic information, such as age, sex, and years of education, were collected using a pre-designed standardized form. Clinical data, including duration of illness, recurrence, medication, and clinical symptom ratings, were obtained from patients in the BPD group. The Young Mania Rating Scale (YMRS), Positive and Negative Syndrome Scale (PANSS), Hamilton Depression Rating Scale (HAMD), Hamilton Anxiety Table (HAMA), and Global Assessment Function (GAF) were applied to evaluate each subject.

### MRI acquisition

MRI scans were obtained for all of the subjects by a skilled medical imaging technician on the Philips Achieva 3.0 T X-series MRI scanner with 8-channel phased array coils at Guangzhou Huiai Hospital. Conventional T1, T2, and blood oxygenation level-dependent (BOLD) images were acquired for each subject. First, 3D T1-weighted volumetric structural images were acquired using a turbo field echo 3D T1 sequence with the following parameters: repetition time (TR) = 8.2 msec, echo time (TE) = 3.8 msec, matrix size = 256 × 256, field of view (FOV) = 250 × 250 mm^2^, number of slices = 188, slice thickness = 1 mm, and inter-slice gap = 0 mm.

Blood oxygenation level-dependent (BOLD) functional images were acquired using a fast field echo (FFE) echo-planar images (EPI) sequence with the following parameters: TR = 2000 msec, TE = 30 msec, flip angle = 30°, slice numbers = 33, matrix size = 64 × 64, FOV = 220 × 220 × 150 mm^3^, inter-slice gap = 0.6 mm, and voxel size = 3.44 × 3.44 × 4 mm. During the fMRI scan time of 523 s, 240 volumes were obtained. Before the scan, the subjects were instructed to “remain still, relaxed, and close eyes but not fall asleep. Try not to think actively.” After the scan, the subjects were asked to confirm that they remained awake during the scanning session.

### Pre-processing and analysis of the structural and functional images

For the structural images, we used the CAT 12 toolbox (http://dbm.neuro.uni-jena.de/cat/) based on the statistic parametric mapping software package (SPM12, http://www.fil.ion.ucl.ac.uk/spm/) in the MATLAB environment (MATLAB 2018b, MathWorks, Natick, MA, USA) to accomplish the data pre-processing and analysis. First, a customized template based on our subjects was created with the segment function in CAT12 and Diffeomorphic Anatomical Registration using Exponentiated Lie (DARTEL) algebra function in SPM 12 [48]. Next, the customized template was used to normalize each subject with a 1.5 × 1.5 × 1.5 mm^3^ voxel size. The normalized images were sent through the standard segmentation and modulation procedure using the default settings in CAT 12 toolbox. Lastly, an 8-mm full-width-half maximum (FWHM) Gaussian smoothing was performed to improve the signal-to-noise ratio. All images were checked for potential image defects or abnormalities.

For the functional images, we used the DPABI_v4.4 (http://www.rfmri.org/dpabi), and SPM12 toolboxes running on MATLAB 2018b were applied to pre-process functional images [49]. First, the first 10 time points were discarded to maintain a steady signal. A total of 230 images for each subject were obtained and corrected for slice timing. Next, using the realign function in DPABI, we corrected the head motion, and anyone with head motions that exceeded 1.5 mm or rotations over 1.5° were excluded. Subsequently, several spurious covariates, including the linear trend of data, white matter, cerebrospinal fluid, and the Friston-24 parameters of head motion, were removed to reduce the effects of scanning time, breathing, and heart beats [50]. Next, the images were normalized to standard Montreal Neurological Institute (MNI) space, resampling to 3 × 3 × 3 mm^3^, based on the previous customized DARTEL template. In the last step of pre-processing, temporal band-pass filtering (0.01–0.08 Hz) was performed to minizine the effects of low-frequency drift and high-frequency physiological noise.

We also used the DPABI toolbox to conduct an H3 ReHo analysis. First, the ReHo map was obtained by calculating Kendall’s correlation coefficient (KCC) for each voxel and the 26 adjacent voxels. Next, the ReHo map was normalized by dividing the averaged KCC of the entire brain. Lastly, a 6-mm FWHM Gaussian smoothing was performed in the normalized ReHo map.

### Feature selection and construction of the Support Vector Machine (SVM)

To obtain more sensitive features and improve the stability and efficiency of the classification in the SVM, a two-sample *t*-test with multiple comparisons was used in the VBM and ReHo statistical analyses [51]. In the VBM analysis, we applied age, sex, education level, and total intracranial volume as nuisance covariates, and the t-map was corrected for multiple comparisons using the Gaussian random field (GRF) theory (voxel-level *p*<0.001, cluster-level *p*<0.05). The grey matter volumes of the clusters showing significant differences were obtained from each subject using the DPABI toolbox. In the ReHo analysis, age, sex, and education level were regarded as nuisance covariates, and the t-map was corrected using the GRF approach (voxel-level *p*<0.01, cluster-level *p*<0.05). The ReHo values of each subject in the clusters showing significant differences were also extracted using the DPABI toolbox. Further, between these regions showed by VBM/Reho analyses, we used LASSO (the least absolute shrinkage selector operator) approach to identify the most informative regions and also reduce the dimensionality of the feature space to avoid over-fitting. Grey matter volumes and ReHo values in each cluster were selected as feature vectors for discrimination and inputted into the SVM to construct the final classification model.

The classification model, which is based on the support vector machine (SVM), was constructed using the LIBSVM soft package in MATLAB environment. All subjects were randomly divided into the training data and test data, where training data were used to learn the difference between groups and build the classification model, and test data were used to evaluate the classification power of the new model. In this process, leave-one-out cross-validation (LOOCV) and grid search methods were applied to ensure the stability and reliability of the model. Next, the accuracy, specificity, sensitivity, and AUC were assessed to comprehensively evaluate the classification model using a permutation test of 5000 times. Also, we compared the performance of the classification model with grey matter volumes alone and ReHo value alone, and with the combination of both grey matter volumes and ReHo.

## Results

### Demographic and clinical information of subjects

In this study, 44 patients diagnosed with BPD and age- and sex-matched 36 healthy controls were recruited. All subjects went through the SCID, and their images were assessed for quality control. None of the subjects were excluded for mental abnormalities, other than BPD, or other defects found in the scans. Demographic and clinical characteristics are summarized in Table 1. In terms of demographics, no significant differences were found in age or gender between the BPD and HC groups (*p* > 0.05), while the length of education was shorter in the BPD group as compared with the HC group. In the BPD group, the age at first onset of the disease was 21.0 ± 5.85, with a disease course of 2.82 ± 1.86 years and recurrence of 1.73 ± 1.19 times. In addition, significant differences in the GAF, HAMA, HAMD, and PANSS scores were found between the BPD group and HC group (*p* < 0.05).

**Table 1:** Demographic and clinical characteristics of subjects in the BPD and HC groups

	BPD	HC	t/ *x*	*P*
Age	23.11 ± 5.15	22.78 ± 2.45	0.3589	0.720
Sex(M/F)	18/26	22/14	3.2323	0.072
Education (years)	12.59 ± 2.94	15.19 ± 1.62	−4.7485	< 0.001
Subtype (I / II)	40/4	–	–	–
Mood status				
depressive	4 (9.1%)	–	–	–
manic	4 (9.1%)	–	–	–
remission	36 (81.8%)	–	–	–
Onset-year	21.3 ± 5.85	–	–	–
Course of disease	2.82 ± 1.86	–	–	–
Recurrence	1.73 ± 1.19	–	–	–
GAF	75.95 ± 14.36	98.86 ± 2.88	−9.410	< 0.001
HAMA	3.64 ± 3.69	0.42 ± 0.65	5.170	< 0.001
HAMD	2.91 ± 3.85	0.39 ± 0.80	3.860	< 0.001
PANSS	41.07 ± 11.87	30.47 ± 1.09	5.335	< 0.001
YOUNG	3.84 ± 7.10	0.03 ± 0.17	3.218	0.002
Medications				
antipsychotics	35 (79.5%)	–	–	–
lithium	18 (41.0%)	–	–	–
valproate	22 (44.7%)	–	–	–
antidepressants	8 (18.2%)	–	–	–

*BPD* bipolar disorder, *HC* healthy controls, *GAF* Global Assessment Function, *HAMA* Hamilton Anxiety Scale, *HAMD* Hamilton Depression Rating Scale, *PANSS* Positive and Negative Syndrome Scale, *YMRS* Young Mania Rating Scale

### Feature selection

**Fig. 1:**
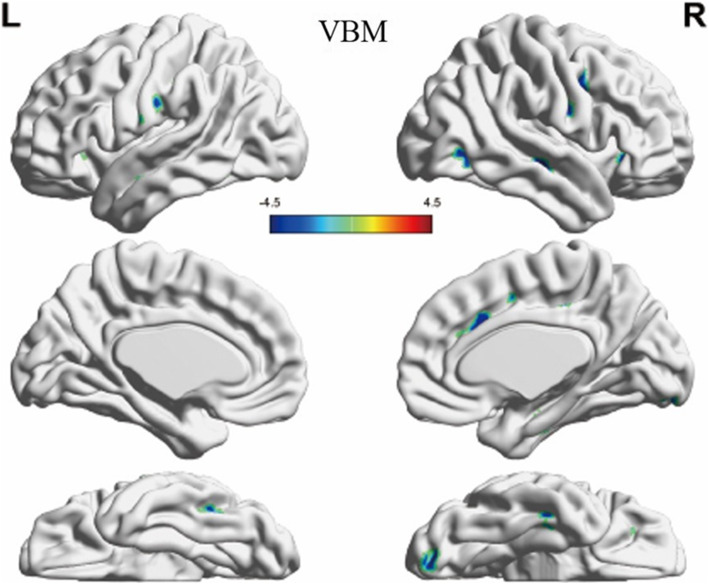
Clusters showing significant differences in between the BPD and HC groups in gray matter volume

**Fig. 2:**
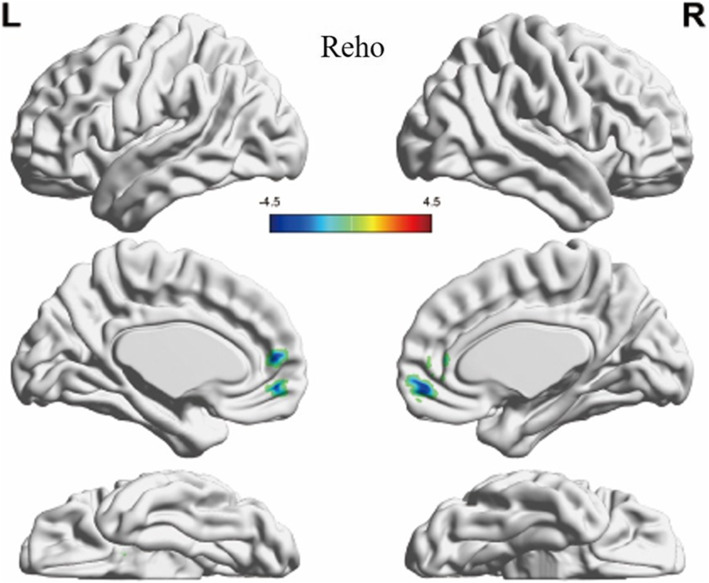
Clusters showing significant differences between the BPD and HC groups in the ReHo values

For the VBM and Reho analysis, a total of 14 clusters showed significant differences between the groups and were extracted. 12 clusters from VBM analyses covered the bilateral inferior frontal gyrus, precentral gyrus, postcentral gyrus, middle occipital gyrus, fusiform and right middle frontal gyrus, cingulate gyrus, anterior cingulate, hippocampus, superior temporal gyrus, lingual gyrus and left limbic lobe, inferior temporal gyrus, and precuneus (GRF-corrected, voxel-level *p* < 0.001, cluster-level *p* < 0.05, Fig. 1). The specific grey matter volumes of these clusters were extracted and shown in Table S1 (Supplementary Materials). Two clusters from Reho analyses covered the right medial frontal gyrus, anterior cingulate, left lentiform nucleus, and putamen (GRF-corrected, voxel-level *p* < 0.01, cluster-level *p* < 0.05, Fig. 2). The ReHo values of the two clusters were extracted and shown in Table S2 (Supplementary Materials). And at last, 8 clusters, 6 from VBM analyses and 2 from Reho analyses, survived from LASSO selection (Table 2).

**Table 2:** Clusters survived from LASSO selection

Cluster number	Brain regions	Peak MNI coordinate			Voxel sizes	T
		x	y	z		
VBM analyses						
1	Right superior temporal gyrus Right hippocampus Right fusiform	45	−20	−14	891	−5.15
2	Right lingual gyrus	15	−89	−14	123	−3.96
3	Left inferior frontal gyrus	−21	35	0	269	−4.23
4	Left Precentral Gyrus Left Postcentral Gyrus	− 54	− 15	28	851	−4.34
5	Right Middle occipital gyrus	35	− 66	29	381	−4.16
6	Left Precuneus Left Middle occipital gyrus	−15	−59	36	489	−4.71
Reho analyses						
1	Left Lentiform Nucleus Left Putamen	−27	6	27	631	4.34
2	Right Medial Frontal Gyrus Right Anterior Cingulate	9	−48	45	648	−4.94

*MNI* Montreal Neurological Institute

### SVM analysis

**Fig. 3:**
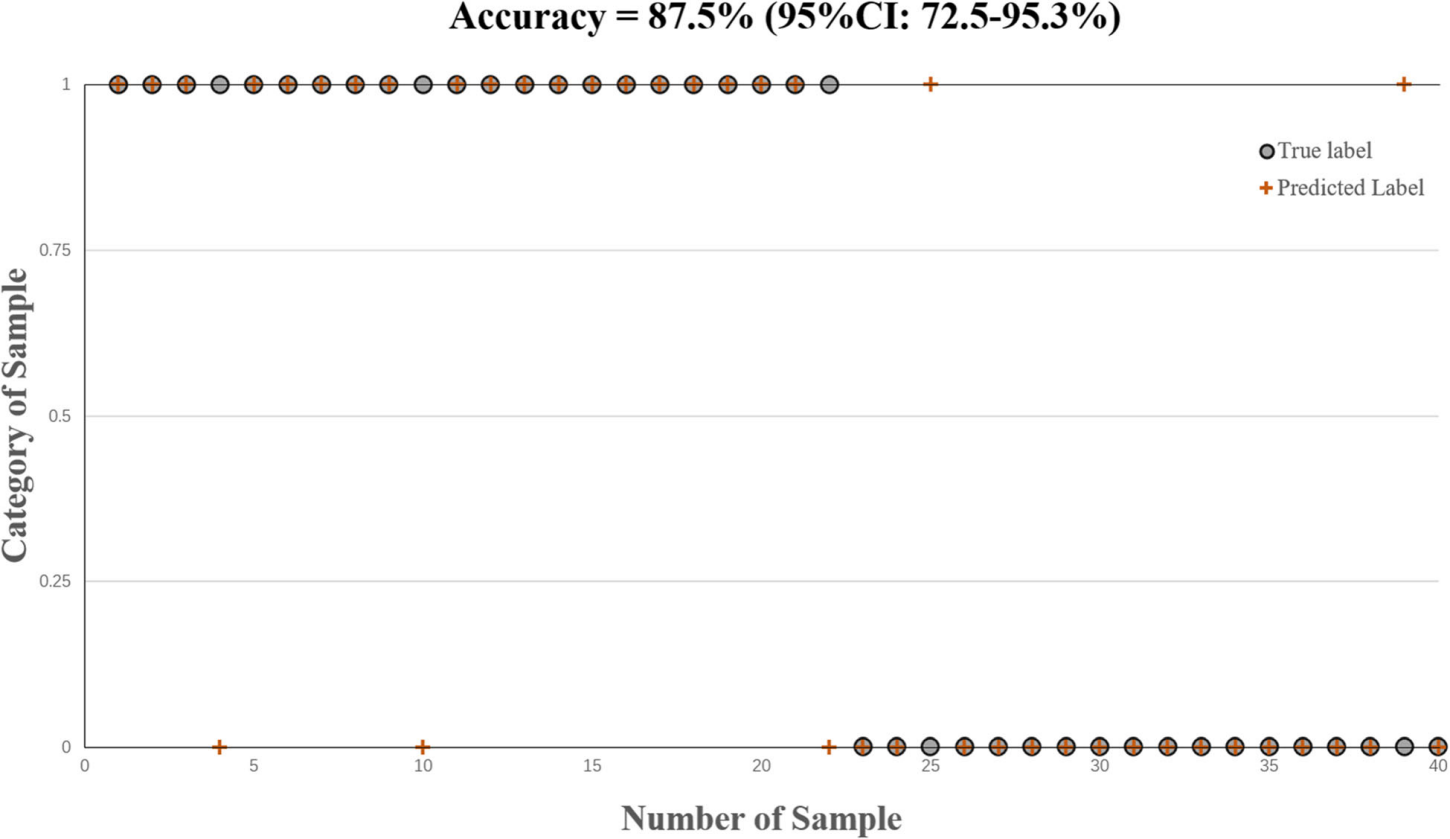
Classification results based on a combination of grey matter volumes and ReHo values

**Fig. 4:**
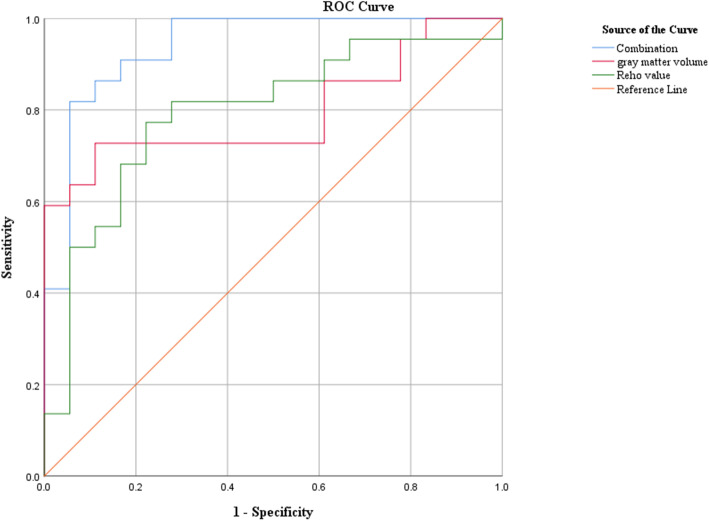
ROC curves showing the performance of the three SVM classifiers

Based on a combination of grey matter volume differences and ReHo values, the trained SVM classifier could correctly identify BPD with an accuracy of 87.5% (95%CI: 72.5–95.3%), sensitivity of 86.4% (95%CI: 64.0–96.4%), and specificity of 88.9% (95%CI: 63.9–98.0%) in the test data (*p* = 0.0022). The specific classification results from the test data are shown in Fig. 3. Based on grey matter volumes alone, the accuracy was reduced to 75% (95%CI: 59.8–85.8%), with a sensitivity of 72.7% (95%CI: 49.6–88.4%) and a specificity of 77.8% (95%CI: 51.9–92.6%), (*p* = 0.0122). Similarly, when based on ReHo values alone, the accuracy was reduced to 77.5% (95%CI: 61.1–88.6%), with increased sensitivity of 77.2% (95%CI: 54.2–91.3%) and specificity of 77.8% (95%CI: 51.9–92.6%), (*p* = 0.0022). As shown in Fig. 4, the area under the receiver operating characteristic (ROC) curves (AUC) of three SVM classifiers were 0.939 (95%CI: 0.865–1.000), 0.795 (95%CI: 0.652–0.939), and 0.793 (95%CI: 0.648–0.938), respectively.

## Discussion

To the best of our knowledge, this is the first study to demonstrate the detection of individual patients with BPD using SVM classifiers based on a combination of ReHo values and grey matter volumes. We constructed the SVM classifier, which could classify BPD with an AUC of 0.949. Our findings showed that the SVM classifier based on a combination of the two performed better than the SVM classifiers based on gray matter volumes alone and ReHo values alone. This result supports our previous hypothesis that the combination of structural and functional MRI could improve the recognition of BPD using an SVM classifier. Similar findings have been detected by other research groups. Using multimodality MRI, several studies have constructed SVM classifiers for the identification of ASD, Alzheimer’s Disease, and schizophrenia [46, 52, 53]. Beyond this, some researchers have combined multimodality MRI with other characteristics of these diseases, such as cerebral spinal fluid, electroencephalography, and eye-tracking [54, 55]. However, markers selection requires careful consideration, as it has been shown that too much data may not improve the power of the SWM classifier in some instances [56]. The finding may be due to the process of over-fitting in machine learning, which may reduce the generalization of the classifier.

In this present study, gray matter volumes and ReHo values were chosen to build the classifiers. Both VBM and ReHo analyses reflect local abnormalities of the brain, in terms of structure and function dimensions independently. In addition, the two approaches are data-driven measurements, which are independent from preconceived assumptions and could make the findings more objective. In the VBM analyses, reduced grey matter volumes were confirmed in multiple cerebral areas, including the frontal lobe, the temporal lobe, the parietal lobe, the limbic system, and the hippocampus. This finding did not go beyond the scope of regions reported by previous literatures. Many researchers have found gray matter shrinkage across extensive frontal regions of inferior frontal gyrus (Stanfield et al., 2009; Lyoo et al., 2014; Wang et al., 2011;), middle frontal gyrus (Li et al., 2011; Brown et al., 2011) and precentral gyrus (Lyoo et al., 2014; Brown et al., 2011) [14, 21, 25, 57, 58]. Inferior and middle frontal gyrus is the primary component of the prefrontal cortex and is involved in aspects of emotional regulatory processes and emotional processing. Abnormal in prefrontal cortex could result in dysfunction in regulating emotion, which is recognized as one of the most fundamental pathophysiology of BPD [59, 60]. Deficits in the gray matter of postcentral gyrus have also been reported in previous studies [61, 62]. Precentral and postcentral gyrus formed the sensorimotor network (SMN). SMN plays a role in the process of emotion regulation, which involves the recognition and feedback of external emotional stimuli [63, 64]. Besides, psychomotor symptoms in patients with BPD are thought to be related with sensorimotor networks [65, 66]. As for the cingulate, anterior cingulate, limbic cortex, and hippocampus, these areas constituted the main body of limbic system. There have been many proofs that show the limbic system involving emotional processing, memory, and executive functioning, as well as its decreases of gray matter in patients with bipolar disorder [19, 23, 67–71]. Besides, we identified decreased gray matter volume in the superior temporal gyrus and inferior temporal gyrus; this finding was consistent with most previous structural MRI studies in BPD [26, 72–74]. Temporal lobe is the brain region related to hearing and vision and thought to play a crucial role in emotional processing, working memory, and social cognition [75, 76].. Several functional MRI studies that found dysfunction of superior temporal gyrus in BD patients with emotional processing impairment provide further support to this deduction. However, the exact mechanism of these abnormalities is still unclear, which be related to auditory processing and involved in superior temporal gyrus [76]. The inferior temporal lobe is a part of the ventral visual pathway related to visual presentation and object recognition. A growing body of evidence suggests that visual impairment may be involved in abnormal pathophysiological processes of bipolar disorder. Researchers have found reduced background effects in visual contrast perception [77]; an autopsy report also revealed lower brain cholesterol levels and a reduction of synapses in the visual cortex [78]; fMRI study further showed dysfunction of visual cortex in patients with bipolar disorder, suggesting that patients have difficulty diverting attention from emotional faces [79]. More ever, some researchers made comparisons between different subtypes of BPD patients. They found that BPD I patients had a decreased superior temporal gyrus gray matter volume than BPD II [74]. Two previous meta-analyses also confirmed decreased gray matter volume in the superior temporal gyrus only in BPD I patients [73, 80]. It can be speculated that deficit in superior temporal gyrus is unique to BPD type I. While most of our patients were diagnosed with BPD type I. This deduction is supported by our findings. However, a large-scale study of cortical abnormalities in BPD patients failed to detect difference between BPD subtypes [81]. More extensive studies are needed to address this inconsistence. The fusiform gyrus and the lingual gyrus, as the same as the inferior temporal lobe, are also involved in visual processing. Gray matter volume reductions in these two regions have been reported in both this present research and previous studies [13, 82–85]. And the precuneus is a part of the parietal lobe, which involves a variety of complex functions, including recollection and memory, integration of information relating to the perception of the environment, episodic memory retrieval, and affective responses pain. Previous studies have reported function deficits of these processes in BPD patients [86, 87]. Our study identified diminished gray matter volume of precuneus in BPD patients; this finding is consistent with functional impairment in BPD patients and a previous meta-analysis of gray matter abnormalities on BPD patients [88]. Besides, contrast to most previous studies, we failed to find any abnormalities in insular in BPD patients. Insular has been regarded as a potential state-related region recently [80]; researchers found that deficits in insular grey matter volume were consistent in all patients with bipolar disorder, except for patients in the euthymic phase [80]. Most of the patients were in remission, which can explain our failure to find any abnormalities in insular in our study. In the ReHo analyses, decreased ReHo value of medial frontal gyrus and anterior cingulate gyrus was found in our study and also have been founded in previous studies [30, 31]. While increases in ReHo were detected in the left putamen and lentiform nucleus in the current study. To our knowledge, this change was first observed in the ReHo analysis of patients with BPD. However, a series of other types of studies implicated this finding. Researchers have found increased gray matter volume and cerebral blood flow in the basal ganglia. Some fMRI studies revealed increased resting-state functional connectivity between basal ganglia and other regions, including the prefrontal cortex, precuneus, and insula [89, 90]. Further, they found that hyperactivity of basal ganglia in bipolar depression patients was positively associated with depressive episode’s duration [89]. This may be explained by biased memory caused by caudate and putamen activity in cognitive models of depression [91]. In general, most of the findings from the VBM and ReHo analyses in the present study have been found in previous studies. These areas cover a wide range of cortical regions and are thoughted to be associated with various aspects of the pathophysiological process of bipolar disorder. However, due to some opposition from other studies [15, 16, 27, 31, 92], direct identification of these abnormalities as biomarkers may be preliminary. Thus, we constructed an SVM classifier with excellent performance in the identification of individual patients with BPD patients with an accuracy of approximately 87.5%. Besides, using the combination of grey matter volumes and ReHo values to construct the SVM classifier, the design performed better than using either gray matter volumes or ReHo values individually. These results support our hypothesis that the combination of structural and functional MRI with an SVM classifier may aid in the detection of BPD in the clinic.

There are some limitations to the present study. It is a small and non-prospective study. Besides, we tested this SVM classifier in the one sample, instead of another independent dataset. These two defects may cause a poor generalization and low confidence of this classifier. Consider this, we performed the leave-one-out cross-validation to improve the reliability and stability of the classifier. In addition, the theoretical basis of the SVM is structural risk minimization instead of empirical risk minimization, which can effectively work with some degree of error and does not require a large sample size. Another key limitation of the current study is potential drug-bias. Due to ethical reasons, all patients in this study were receiving medications. Previous studies have reported increased gray matter in paracentral gyrus and superior parietal gyrus caused by lithium and reduced gray matter in visual cortex caused by antiepileptics [81]. In our study, approximately 80% of BPD patients were prescribed with mood stabilizers, and more than half of them were prescribed with valproate. Deficits in ventral visual pathway also were found in present study. Hence, it can be difficult to distinguish whether these deficits are medication-induced or abnormalities detected in the disease itself. A further distinction may be needed using unmedicated patients with long-term follow-ups. In addition, illness duration is also an important confounder. Hibar et al. (2017) have reported that long duration of illness could lead to reduced gray matter in pericalcarine gyrus, anterior cingulate gyrus, cuneus and increased gray matter in entorhinal gyrus. However, the illness duration of BPD patients in a previous study was much longer than that in our study, more than 10 years and less than 3 years, respectively. Thus, the effect of illness duration in our study was relatively weak. Lastly, this study focused on grey matter volumes and ReHo values as discriminant features to construct the SVM classifier, yet these may not provide a comprehensive assessment of the brain. More suitable neuroimaging biomarkers, such as those to detect changes in white matter microstructures and cerebral blood flow, should be considered in future studies.

## Conclusions

In this study, we have shown grey matter volumes and ReHo values, as the discriminate features, could be used to conduct SVM classifiers and recognize patients with BPD at the individual level. Compared with the single-modality MRI, the combination of structural and functional MRI data could be of added value in the construction of SVM classifiers for the accurate detection of BPD.

## Supplementary information

**Additional file 1: Table S1.** Grey matter volume of clusters showing significant differences between groups between the BPD and HC groups. **Table S2.** Reho value of clusters showing significant differences between between the BPD and HC groups.

## Data Availability

The data used to support the findings of this study are available from the corresponding author upon request.

## Abbreviations

BPD: Bipolar disorder
MDD: Major depressive disorder
ADHD: Attention deficit and hyperactivity disorder
HC: Healthy controls
DSM-IV: The Diagnostic and Statistical Manual of Mental Disorders 4th Edition
SCID: Structured clinical interview for DSM-IV
YMRS: Young Mania Rating Scale
PANSS: Positive and Negative Syndrome Scale
HAMD: Hamilton Depression Rating Scale
HAMA: Hamilton Anxiety Table
GAF: Global Assessment Function
SVM: Support vector machine
AUC: Area Under Curve
ROC: Receiver operating characteristic curve
MRI: Magnetic resonance imaging
FFE: Fast field echo
EPI: Echo-planar images
TR: Repetition time
TE: Echo time
FOV: Field of view (FOV)
fMRI: Functional MRI fMRI
KCC: Kendall’s coefficient of concordance
BOLD: Blood oxygen level-dependent
VBM: Voxel-based morphometry
ReHo: Regional homogeneity
DARTEL: Diffeomorphic Anatomical Registration using Exponentiated Lie
FWHM: Full-width-half maximum
MNI: Montreal Neurological Institute space
GRF: Gaussian random field
LASSO: The least absolute shrinkage selector operator
SMN: Sensorimotor network
PFC: Prefrontal cortex
