# Supplementary material for: Identification of bipolar disorder using a combination of multimodality magnetic resonance imaging and machine learning techniques
Source: BMC Psychiatry. 2020 Oct 6;20:488. doi: 10.1186/s12888-020-02886-5 (PMC7542439; doi:10.1186/s12888-020-02886-5)
Supplement: Supplementary file 1 — Additional file 1: Table S1. Grey matter volume of clusters showing significant differences between groups between the BPD and HC groups. Table S2. Reho value of clusters showing significant differences between between the BPD and HC groups. [file 12888_2020_2886_MOESM1_ESM.pdf]

Table S1 Grey matter volume of clusters showing significant differences between groups between the BPD and HC groups

| Subjects | cluster 1 | cluster 2 | cluster 3 | cluster 4 | cluster 5 | cluster 6 | cluster 7 | cluster 8 |
|---|---|---|---|---|---|---|---|---|
| BP 1 | 0.43025 | 0.22726 | 0.3609 | 0.25269 | 0.37478 | 0.31513 | 0.33786 | 0.23691 |
| BP 2 | 0.38382 | 0.22152 | 0.37783 | 0.30871 | 0.38854 | 0.39197 | 0.339 | 0.28952 |
| BP 3 | 0.45561 | 0.30499 | 0.48807 | 0.29106 | 0.39273 | 0.43441 | 0.37739 | 0.27603 |
| BP 4 | 0.35711 | 0.21626 | 0.33422 | 0.27267 | 0.39725 | 0.31369 | 0.28332 | 0.21173 |
| BP 5 | 0.38907 | 0.24843 | 0.38813 | 0.26617 | 0.35634 | 0.35636 | 0.35928 | 0.31199 |
| BP 6 | 0.44254 | 0.2587 | 0.50459 | 0.30856 | 0.4246 | 0.38815 | 0.36216 | 0.34392 |
| BP 7 | 0.39803 | 0.23564 | 0.36614 | 0.25286 | 0.41959 | 0.37786 | 0.37044 | 0.29112 |
| BP 8 | 0.51435 | 0.36027 | 0.59283 | 0.33444 | 0.45285 | 0.42537 | 0.40536 | 0.38887 |
| BP 9 | 0.38915 | 0.21135 | 0.30372 | 0.27037 | 0.32244 | 0.30636 | 0.28729 | 0.23121 |
| BP 10 | 0.38046 | 0.2446 | 0.35809 | 0.27138 | 0.36091 | 0.3174 | 0.32933 | 0.25798 |
| BP 11 | 0.3478 | 0.21001 | 0.34382 | 0.26298 | 0.36214 | 0.30169 | 0.35613 | 0.2125 |
| BP 12 | 0.46556 | 0.31593 | 0.49982 | 0.32371 | 0.44775 | 0.37161 | 0.42311 | 0.31731 |
| BP 13 | 0.38733 | 0.1883 | 0.42253 | 0.29654 | 0.38177 | 0.31198 | 0.30919 | 0.33528 |
| BP 14 | 0.47283 | 0.34468 | 0.55515 | 0.31381 | 0.44874 | 0.3209 | 0.37184 | 0.36366 |
| BP 15 | 0.40522 | 0.2902 | 0.43669 | 0.28903 | 0.38114 | 0.36192 | 0.37503 | 0.30239 |
| BP 16 | 0.26207 | 0.28312 | 0.2929 | 0.24722 | 0.3018 | 0.29245 | 0.24318 | 0.22386 |
| BP 17 | 0.37749 | 0.21157 | 0.35564 | 0.2583 | 0.36197 | 0.31678 | 0.35016 | 0.25611 |
| BP 18 | 0.45946 | 0.20293 | 0.46866 | 0.2905 | 0.37992 | 0.41052 | 0.40981 | 0.27382 |
| BP 19 | 0.38339 | 0.23246 | 0.41551 | 0.35261 | 0.40649 | 0.41464 | 0.29695 | 0.2803 |
| BP 20 | 0.36959 | 0.29338 | 0.38006 | 0.28432 | 0.3798 | 0.33126 | 0.35183 | 0.27426 |
| BP 21 | 0.32106 | 0.22653 | 0.41013 | 0.21841 | 0.33338 | 0.25616 | 0.25244 | 0.20704 |
| BP 22 | 0.47966 | 0.30148 | 0.46282 | 0.32549 | 0.4864 | 0.41204 | 0.38066 | 0.33968 |
| BP 23 | 0.42165 | 0.19519 | 0.43504 | 0.29272 | 0.46415 | 0.38606 | 0.37233 | 0.31038 |
| BP 24 | 0.39479 | 0.26785 | 0.41401 | 0.2997 | 0.41728 | 0.30935 | 0.36522 | 0.29094 |
| BP 25 | 0.35032 | 0.20644 | 0.34945 | 0.25646 | 0.32076 | 0.24747 | 0.2946 | 0.20992 |
| BP 26 | 0.43734 | 0.22197 | 0.46585 | 0.31033 | 0.39984 | 0.33429 | 0.32905 | 0.30735 |
| BP 27 | 0.4181 | 0.3597 | 0.42225 | 0.29194 | 0.37655 | 0.31739 | 0.32959 | 0.32464 |
| BP 28 | 0.38045 | 0.28439 | 0.38178 | 0.2591 | 0.35729 | 0.34356 | 0.32151 | 0.25188 |
| BP 29 | 0.36345 | 0.20462 | 0.35574 | 0.27721 | 0.37732 | 0.30122 | 0.24653 | 0.24475 |
| BP 30 | 0.48115 | 0.32249 | 0.47105 | 0.39545 | 0.54822 | 0.42228 | 0.39979 | 0.34709 |
| BP 31 | 0.4499 | 0.18192 | 0.36313 | 0.25404 | 0.32616 | 0.28788 | 0.28736 | 0.2281 |
| BP 32 | 0.36567 | 0.34417 | 0.36591 | 0.27945 | 0.40385 | 0.3428 | 0.37141 | 0.27268 |
| BP 33 | 0.34294 | 0.19193 | 0.31757 | 0.25919 | 0.35238 | 0.34458 | 0.32282 | 0.24211 |
| BP 34 | 0.47854 | 0.26163 | 0.42551 | 0.41596 | 0.51395 | 0.41811 | 0.40072 | 0.28494 |
| BP 35 | 0.38933 | 0.22439 | 0.37507 | 0.30499 | 0.33907 | 0.33469 | 0.37757 | 0.28681 |
| BP 36 | 0.42118 | 0.31271 | 0.4024 | 0.28452 | 0.41202 | 0.30755 | 0.32713 | 0.26804 |
| BP 37 | 0.4123 | 0.22704 | 0.38073 | 0.25228 | 0.35775 | 0.3341 | 0.2957 | 0.26382 |
| BP 38 | 0.45753 | 0.28816 | 0.43307 | 0.33064 | 0.46555 | 0.38837 | 0.37299 | 0.3327 |
| BP 39 | 0.43239 | 0.29364 | 0.41085 | 0.33218 | 0.41821 | 0.37862 | 0.34005 | 0.29913 |
| BP 40 | 0.30955 | 0.23134 | 0.40848 | 0.23467 | 0.3451 | 0.35617 | 0.29598 | 0.25494 |
| BP 41 | 0.42617 | 0.25203 | 0.33391 | 0.30616 | 0.41475 | 0.32923 | 0.30146 | 0.29731 |
| BP 42 | 0.53524 | 0.34089 | 0.48836 | 0.36647 | 0.51801 | 0.40119 | 0.41171 | 0.35126 |
| BP 43 | 0.38691 | 0.27272 | 0.48848 | 0.24837 | 0.39716 | 0.32602 | 0.30655 | 0.25176 |
| BP 44 | 0.3994 | 0.25567 | 0.38844 | 0.3503 | 0.46175 | 0.40822 | 0.41918 | 0.33419 |
| HC 1 | 0.41905 | 0.24939 | 0.4842 | 0.30706 | 0.43097 | 0.35607 | 0.36659 | 0.31559 |
| HC 2 | 0.52396 | 0.28707 | 0.50495 | 0.31561 | 0.49346 | 0.41765 | 0.40005 | 0.29593 |
| HC 3 | 0.43649 | 0.25592 | 0.41066 | 0.2969 | 0.4076 | 0.32275 | 0.35782 | 0.28267 |
| HC 4 | 0.53432 | 0.34845 | 0.49249 | 0.34147 | 0.47433 | 0.46526 | 0.41876 | 0.32703 |
| HC 5 | 0.50668 | 0.29574 | 0.50517 | 0.39068 | 0.50393 | 0.46622 | 0.42188 | 0.37976 |
| HC 6 | 0.5331 | 0.24129 | 0.43938 | 0.43432 | 0.51991 | 0.50848 | 0.40887 | 0.38284 |
| HC 7 | 0.51711 | 0.32575 | 0.51059 | 0.31845 | 0.45981 | 0.492 | 0.3966 | 0.37081 |

| | | | | | | | | |
|---|---|---|---|---|---|---|---|---|
| HC 8 | 0.49859 | 0.24218 | 0.47032 | 0.36387 | 0.46843 | 0.37592 | 0.45306 | 0.36526 |
| HC 9 | 0.39634 | 0.30652 | 0.56905 | 0.30523 | 0.41078 | 0.35307 | 0.35395 | 0.27221 |
| HC 10 | 0.42894 | 0.26966 | 0.39068 | 0.30044 | 0.40574 | 0.40926 | 0.34386 | 0.27943 |
| HC 11 | 0.5011 | 0.3465 | 0.55519 | 0.29221 | 0.46073 | 0.35712 | 0.39567 | 0.30675 |
| HC 12 | 0.48434 | 0.29896 | 0.49561 | 0.35845 | 0.46411 | 0.42598 | 0.37296 | 0.30398 |
| HC 13 | 0.44447 | 0.40666 | 0.45874 | 0.31425 | 0.40107 | 0.34824 | 0.35586 | 0.30475 |
| HC 14 | 0.46649 | 0.31306 | 0.51384 | 0.37679 | 0.52501 | 0.50227 | 0.43443 | 0.40446 |
| HC 15 | 0.40597 | 0.27725 | 0.41466 | 0.33817 | 0.44582 | 0.42073 | 0.39096 | 0.33293 |
| HC 16 | 0.46525 | 0.2733 | 0.41794 | 0.30957 | 0.40609 | 0.38852 | 0.38709 | 0.32304 |
| HC 17 | 0.42828 | 0.32281 | 0.45309 | 0.34934 | 0.46158 | 0.4458 | 0.38301 | 0.38414 |
| HC 18 | 0.43891 | 0.4059 | 0.51315 | 0.29038 | 0.40338 | 0.32989 | 0.39025 | 0.31195 |
| HC 19 | 0.44112 | 0.24027 | 0.412 | 0.28864 | 0.41803 | 0.34243 | 0.34062 | 0.32085 |
| HC 20 | 0.47376 | 0.35798 | 0.48561 | 0.29458 | 0.3931 | 0.41172 | 0.39381 | 0.31363 |
| HC 21 | 0.51533 | 0.30537 | 0.51966 | 0.35577 | 0.46797 | 0.52 | 0.46641 | 0.39772 |
| HC 22 | 0.46991 | 0.3686 | 0.43859 | 0.29652 | 0.41835 | 0.36359 | 0.3584 | 0.29301 |
| HC 23 | 0.49062 | 0.33298 | 0.47057 | 0.33007 | 0.46102 | 0.42452 | 0.39085 | 0.3881 |
| HC 24 | 0.48518 | 0.32077 | 0.43299 | 0.28732 | 0.43499 | 0.40663 | 0.39175 | 0.372 |
| HC 25 | 0.3686 | 0.29839 | 0.42108 | 0.33864 | 0.44963 | 0.3489 | 0.36536 | 0.28936 |
| HC 26 | 0.45472 | 0.22522 | 0.49128 | 0.33195 | 0.42104 | 0.43572 | 0.35969 | 0.37401 |
| HC 27 | 0.38754 | 0.30333 | 0.39772 | 0.30062 | 0.45166 | 0.36329 | 0.35057 | 0.32459 |
| HC 28 | 0.42219 | 0.3129 | 0.44099 | 0.31314 | 0.39078 | 0.38213 | 0.33558 | 0.26465 |
| HC 29 | 0.43533 | 0.27276 | 0.48592 | 0.30888 | 0.43816 | 0.36198 | 0.38677 | 0.37144 |
| HC 30 | 0.50624 | 0.35527 | 0.51266 | 0.3618 | 0.43524 | 0.45744 | 0.42159 | 0.32195 |
| HC 31 | 0.44765 | 0.37335 | 0.56833 | 0.37348 | 0.50204 | 0.50031 | 0.43759 | 0.39806 |
| HC 32 | 0.56976 | 0.28417 | 0.6011 | 0.38112 | 0.48349 | 0.42504 | 0.39295 | 0.32401 |
| HC 33 | 0.4066 | 0.24502 | 0.4287 | 0.32422 | 0.38856 | 0.37376 | 0.32557 | 0.26543 |
| HC 34 | 0.53724 | 0.26985 | 0.4678 | 0.35449 | 0.43348 | 0.44417 | 0.41018 | 0.35095 |
| HC 35 | 0.36429 | 0.19372 | 0.34737 | 0.26304 | 0.36252 | 0.29048 | 0.35323 | 0.2088 |
| HC 36 | 0.43667 | 0.36008 | 0.38027 | 0.26636 | 0.45142 | 0.39913 | 0.35438 | 0.32647 |

| cluster 9 | cluster 10 | cluster 11 | cluster 12 |
|---|---|---|---|
| 0.20897 | 0.31816 | 0.30733 | 0.57513 |
| 0.23229 | 0.387 | 0.25185 | 0.58583 |
| 0.26219 | 0.41236 | 0.33016 | 0.57496 |
| 0.24834 | 0.3914 | 0.40705 | 0.49869 |
| 0.21916 | 0.34756 | 0.29691 | 0.62938 |
| 0.30202 | 0.45677 | 0.36888 | 0.58844 |
| 0.27753 | 0.425 | 0.37544 | 0.51789 |
| 0.32536 | 0.42205 | 0.39916 | 0.77843 |
| 0.21668 | 0.37882 | 0.23679 | 0.56457 |
| 0.21698 | 0.30729 | 0.31526 | 0.49921 |
| 0.2138 | 0.42996 | 0.4332 | 0.5891 |
| 0.27693 | 0.52523 | 0.39554 | 0.70279 |
| 0.24293 | 0.39793 | 0.41058 | 0.49732 |
| 0.30267 | 0.57122 | 0.41145 | 0.6 |
| 0.224 | 0.30194 | 0.34828 | 0.46969 |
| 0.17691 | 0.26878 | 0.24143 | 0.44565 |
| 0.21164 | 0.29163 | 0.2523 | 0.51087 |
| 0.23984 | 0.46605 | 0.37867 | 0.66013 |
| 0.25909 | 0.4733 | 0.37401 | 0.65225 |
| 0.20937 | 0.46874 | 0.34687 | 0.56027 |
| 0.19149 | 0.27109 | 0.31132 | 0.52982 |
| 0.27739 | 0.44253 | 0.45376 | 0.7687 |
| 0.24551 | 0.41627 | 0.29611 | 0.6375 |
| 0.23397 | 0.3797 | 0.32927 | 0.55783 |
| 0.23169 | 0.34733 | 0.26762 | 0.49714 |
| 0.26026 | 0.34601 | 0.25325 | 0.56918 |
| 0.23755 | 0.58035 | 0.38062 | 0.584 |
| 0.22114 | 0.35562 | 0.27368 | 0.52577 |
| 0.21175 | 0.33891 | 0.28317 | 0.48204 |
| 0.29038 | 0.41553 | 0.37763 | 0.64727 |
| 0.19232 | 0.4698 | 0.34065 | 0.51084 |
| 0.26989 | 0.33372 | 0.32126 | 0.65635 |
| 0.20882 | 0.29099 | 0.21954 | 0.4872 |
| 0.27059 | 0.40727 | 0.32789 | 0.66072 |
| 0.22693 | 0.42077 | 0.45558 | 0.59233 |
| 0.23578 | 0.40624 | 0.29568 | 0.6537 |
| 0.22122 | 0.29417 | 0.37746 | 0.51256 |
| 0.25464 | 0.57433 | 0.3775 | 0.73313 |
| 0.26214 | 0.38515 | 0.29449 | 0.55301 |
| 0.19768 | 0.31359 | 0.19683 | 0.53117 |
| 0.25746 | 0.40568 | 0.3803 | 0.559 |
| 0.30814 | 0.42597 | 0.45333 | 0.6528 |
| 0.24092 | 0.40855 | 0.39456 | 0.57227 |
| 0.263 | 0.51351 | 0.42634 | 0.63223 |
| 0.25702 | 0.46995 | 0.43754 | 0.59329 |
| 0.28783 | 0.54379 | 0.39061 | 0.65958 |
| 0.22855 | 0.37431 | 0.32891 | 0.55287 |
| 0.25604 | 0.31941 | 0.2469 | 0.56474 |
| 0.3055 | 0.53606 | 0.47441 | 0.68634 |
| 0.32673 | 0.46455 | 0.40882 | 0.70571 |
| 0.26539 | 0.40995 | 0.46495 | 0.68693 |

| | | | |
|---|---|---|---|
| 0.29931 | 0.53251 | 0.43408 | 0.65834 |
| 0.27448 | 0.51006 | 0.42213 | 0.67107 |
| 0.25072 | 0.35929 | 0.31887 | 0.57499 |
| 0.29446 | 0.42086 | 0.35832 | 0.67434 |
| 0.26229 | 0.40862 | 0.38272 | 0.66901 |
| 0.27391 | 0.47102 | 0.348 | 0.60815 |
| 0.34271 | 0.68868 | 0.74107 | 0.69859 |
| 0.29439 | 0.50337 | 0.35536 | 0.65053 |
| 0.25743 | 0.5461 | 0.38636 | 0.571 |
| 0.32135 | 0.52348 | 0.54722 | 0.66096 |
| 0.24279 | 0.53953 | 0.40875 | 0.72453 |
| 0.25391 | 0.53784 | 0.42387 | 0.68689 |
| 0.24336 | 0.59188 | 0.51916 | 0.59931 |
| 0.32537 | 0.41969 | 0.47069 | 0.63364 |
| 0.23683 | 0.36142 | 0.40274 | 0.64558 |
| 0.26451 | 0.44569 | 0.41338 | 0.76109 |
| 0.25658 | 0.39262 | 0.46963 | 0.67145 |
| 0.2395 | 0.51038 | 0.44156 | 0.53185 |
| 0.2696 | 0.49458 | 0.44201 | 0.64912 |
| 0.29951 | 0.4912 | 0.42939 | 0.62074 |
| 0.24702 | 0.44551 | 0.34312 | 0.60619 |
| 0.29314 | 0.49394 | 0.36708 | 0.58597 |
| 0.26754 | 0.60213 | 0.43627 | 0.6087 |
| 0.38516 | 0.51524 | 0.52506 | 0.86835 |
| 0.29599 | 0.54446 | 0.3946 | 0.72914 |
| 0.25503 | 0.49899 | 0.41828 | 0.62036 |
| 0.30382 | 0.38551 | 0.33847 | 0.64338 |
| 0.21282 | 0.34092 | 0.23715 | 0.48538 |
| 0.27702 | 0.34045 | 0.33859 | 0.65738 |

Table S2 Reho value of clusters showing significant differences between between the BPD and HC groups

| Subjects | cluster1 | cluster 2 |
|---|---|---|
| BP 1 | 0.85271 | 1.169402 |
| BP 2 | 0.742965 | 1.279987 |
| BP 3 | 0.855027 | 1.354173 |
| BP 4 | 0.811142 | 1.35747 |
| BP 5 | 0.913037 | 1.153537 |
| BP 6 | 0.840604 | 1.375421 |
| BP 7 | 0.803765 | 1.25892 |
| BP 8 | 0.868395 | 1.280978 |
| BP 9 | 0.84456 | 1.27296 |
| BP 10 | 0.872648 | 1.339584 |
| BP 11 | 0.842461 | 1.241976 |
| BP 12 | 0.912466 | 1.249289 |
| BP 13 | 0.809859 | 1.347494 |
| BP 14 | 0.917068 | 1.275044 |
| BP 15 | 0.860036 | 1.274404 |
| BP 16 | 0.842561 | 1.191816 |
| BP 17 | 0.860547 | 1.171986 |
| BP 18 | 0.904556 | 1.151592 |
| BP 19 | 0.870384 | 1.127453 |
| BP 20 | 0.826819 | 1.192816 |
| BP 21 | 0.839191 | 1.278091 |
| BP 22 | 0.825884 | 1.313388 |
| BP 23 | 0.877308 | 1.314873 |
| BP 24 | 0.827886 | 1.140463 |
| BP 25 | 0.778863 | 1.274679 |
| BP 26 | 0.826006 | 1.300686 |
| BP 27 | 0.8546 | 1.372037 |
| BP 28 | 0.970088 | 1.188285 |
| BP 29 | 0.882984 | 1.166331 |
| BP 30 | 0.929674 | 1.18964 |
| BP 31 | 0.914817 | 1.345324 |
| BP 32 | 0.821396 | 1.181489 |
| BP 33 | 0.792478 | 1.286547 |
| BP 34 | 0.884424 | 1.218914 |
| BP 35 | 0.898424 | 1.196624 |
| BP 36 | 0.830875 | 1.285171 |
| BP 37 | 0.839387 | 1.155854 |
| BP 38 | 0.879035 | 1.249394 |
| BP 39 | 0.846955 | 1.177587 |
| BP 40 | 0.769359 | 1.214837 |
| BP 41 | 0.837813 | 1.157573 |
| BP 42 | 0.895085 | 1.214872 |
| BP 43 | 0.747486 | 1.25153 |
| BP 44 | 0.82861 | 1.313149 |
| HC 1 | 0.807629 | 1.421241 |
| HC 2 | 0.747797 | 1.376536 |
| HC 3 | 0.878531 | 1.239498 |
| HC 4 | 0.768265 | 1.341779 |
| HC 5 | 0.749024 | 1.315095 |
| HC 6 | 0.729647 | 1.258139 |
| HC 7 | 0.65723 | 1.43295 |

| | | |
|---|---|---|
| HC 8 | 0.760751 | 1.329688 |
| HC 9 | 0.800856 | 1.336849 |
| HC 10 | 0.751114 | 1.278918 |
| HC 11 | 0.712382 | 1.361221 |
| HC 12 | 0.764752 | 1.476261 |
| HC 13 | 0.780015 | 1.402038 |
| HC 14 | 0.734638 | 1.417674 |
| HC 15 | 0.761048 | 1.371912 |
| HC 16 | 0.735081 | 1.456045 |
| HC 17 | 0.747384 | 1.462457 |
| HC 18 | 0.793034 | 1.369975 |
| HC 19 | 0.743683 | 1.449751 |
| HC 20 | 0.734356 | 1.334721 |
| HC 21 | 0.746193 | 1.36458 |
| HC 22 | 0.781185 | 1.320665 |
| HC 23 | 0.828343 | 1.368091 |
| HC 24 | 0.81553 | 1.379738 |
| HC 25 | 0.795441 | 1.332298 |
| HC 26 | 0.863406 | 1.353309 |
| HC 27 | 0.837867 | 1.279123 |
| HC 28 | 0.787267 | 1.52232 |
| HC 29 | 0.850261 | 1.315342 |
| HC 30 | 0.770692 | 1.425654 |
| HC 31 | 0.698058 | 1.418489 |
| HC 32 | 0.765077 | 1.418626 |
| HC 33 | 0.758171 | 1.435211 |
| HC 34 | 0.808667 | 1.344383 |
| HC 35 | 0.788192 | 1.30765 |
| HC 36 | 0.757086 | 1.301977 |
